# Idiopathic thrombocytopenic purpura with brain abscess caused by *Nocardia farcinica* diagnosed using metagenomics next-generation sequencing of the cerebrospinal fluid: a case report

**DOI:** 10.1186/s12879-021-06071-1

**Published:** 2021-04-23

**Authors:** Chaojun Zhou, Kai Wang, Hanrong Li, Xiaobo Zhang

**Affiliations:** Department of Neurology, The First People’s Hospital of Changde City, Changde, Hunan China

**Keywords:** *Nocardia farcinica*, Brain abscess, Next-generation sequencing, Cerebrospinal fluid

## Abstract

**Background:**

Brain abscesses caused by *Nocardia farcinica* are rare, and mostly occur in immunocompromised individuals. Rapid and accurate diagnosis of *nocardiosis* is challenging. Due to the inadequate performance of conventional diagnostic methods for *Nocardia* infection, metagenomics next-generation sequencing (mNGS) of cerebrospinal fluid (CSF) has the potential to improve the diagnosis intracranial nocardiosis.

**Case presentation:**

We report a case of 50-year-old man with brain abscess caused by *Nocardia farcinica*. The patient had a idiopathic thrombocytopenic purpura complication that required long-term methylprednisolone administration. His chest image showed multiple lesions, which had been misdiagnosed as lung cancer, and his head image showed multiple intracranial metastases. No pathogen was detected in routine examinations including blood culture, sputum culture and traditional culture methods of cerebrospinal fluid. In order to accurately identify the pathogen, mNGS was used to detect *Nocardia* in CSF. Although the patient’s condition improved after using sensitive antibiotics, he transferred to the local hospital for treatment because of many complicated diseases and family financial limitations.

**Conclusion:**

This case highlights the value of mNGS in the diagnosis of *Nocardia* brain abscess, and emphasizes the inadequate sensitivity of conventional diagnostic methods for *Nocardia* infection. Using mNGS can facilitate early and accurate detection of Norcadia-associated of meningitis in immunocompromised patients, thereby reducing unnecessary use of antibiotics and reducing mortality of the disease.

## Background

Nocardia farcinica is a bacterium that widely exists in soil, sea water, fresh water, and dust. It is a Gram-positive with some acid resistance and positive acid-fast staining. It enters the human body through the respiratory tract and can cause disseminated infections of central nervous system (CNS) [1]. *Nocardia* is an opportunistic pathogen. The CNS infection caused by *Nocardia* often occurs in immunocompromised individuals, such as in cases of organ transplantation, leukemia, HIV infection, autoimmune diseases and long-term use of steroids. It is easy to spread through the skin or lung, causing serious systemic infection [2]. Epilepsy and focal neurological dysfunction are the most common clinical manifestations of *Nocardia* brain abscess, but the condition has no typical pathognomonic signs or symptoms to facilitate a quick presumptive diagnosis.

*Nocardia* brain abscess is the characteristic signs of a CNS infection, accounting for 1% ~ 2% of all intracranial abscesses. Among all bacterial brain abscesses, *Nocardia* brain abscess accounts for the highest mortality. The high mortality is mainly attributed to the severity of underlying disease, difficulties in identifying the pathogen, and its inherent resistance to antibiotics, leading to inappropriate or late initiation of therapy [3].^.^However, *Nocardia* infection of the CNS is very rare clinically. The current routine diagnosis methods are not good for the diagnosis of *Nocardia* infection. mNGS successfully identified *Nocardia farcinica*, which has a crucial impact on the prognosis of patients [4].

Here we present a case previously misdiagnosed as lung cancer with brain metastasis but later confirmed, using mNGS, to be a Nocardia farcinica brain abscess.

## Case presentation

A 50-year-old male patient with idiopathic thrombocytopenic purpura and pneumonia thrice admitted to the respiratory department of our hospital over a period of 4 months in early 2020. Lung CT scan showed multiple lung masses. The pathological section on 03-Apr-2020 showed pyogranulomatous inflammation. However, because the patient was not treated systematically, the pneumonia had not been eradicated.

**Fig. 1:**
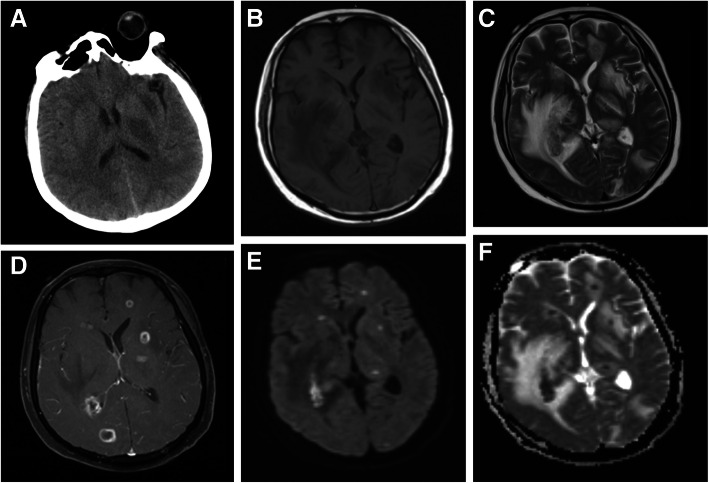
Imaging changes of the patient’s brain. CT (**a**) and MRI (**b**-**f**) indicates brain abscess

The patient had a history of atrial fibrillation and long-term use of methylprednisolone. A month later, he was admitted to the hospital with headache and vomiting for 4 days. Routine blood analysis at the emergency department showed a white blood cell (WBC) count of 10.68 × 10^9/L, N%: 74.8%. A head CT scan showed multiple low-density shadows without bleeding (Fig. 1).

After entering our department, the patient developed fever, with a temperature of nearly 39.0 °C, and accompanied by disturbance of consciousness but without seizures. Physical examination of his nervous system showed shallow coma and left hemiplegia with some signs of meningitis. His Glasgow Coma Scale (GCS) score was 7.

On the 2nd day following admission, a lumbar puncture was performed, and it showed turbid CSF. The CSF pressure was 380 mmH_2_O (normal range,80-180mmH2O) and the color of the CSF was light yellow, with slight turbidity. The WBC count was 350/μL. Analysis of the CSF revealed that the levels of glucose, chloride and protein were 5.61 mmol/L (normal range, 2.5–4.5 mmol/L), 118.2 mmol/L (normal range, 120–132 mmol/L). and 2087 mg/L (normal range, 150–450 mg /L) respectively. CSF stain including Gram stain, ink stain and acid-fast stain were negative.

Two days after admission, Magnetic resonance imaging (MRI) of the brain revealed multiple irregular lesions in the right temporal lobe and bilateral basal ganglia with significant edema and local mass effect. Following contrast, most of these lesions enhanced in a smooth ring fashion (Fig. 1). Magnetic resonance venography (MRV) showed no cerebral venous sinus thrombosis. The procalcitonin (PCT) was 0.58 ng/ml and Endotoxin was 0.0444EU/ml. But the erythrocyte sedimentation rate (ESR; normal range 0 to 20 mm per hour) and C-reactive protein (CRP) level (normal range, < 10 mg/L) were elevated to 79 mm/h and 228 mg/l respectively. After treatment with meropenem and vancomycin for 3 days, the clinical symptoms of the patient did not improve. Serological tests and traditional CSF culture also did not detect possible infection aetiologies. Additionally, antineutrophil cytoplasmic antibody, syphilis antibody, HIV antibody and tumor markers of serum were in normal range. (1, 3)-β-D-glucan and galactomannan testing were also negative.

**Fig. 2:**
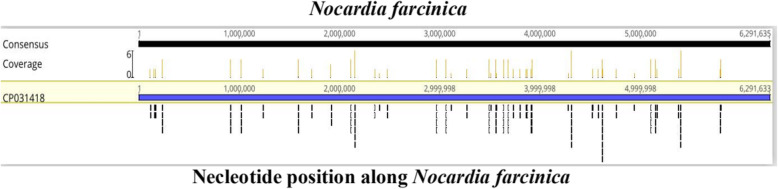
mNGS results of pathogen identification: mNGS analysis of the CSF confirmed the presence of *Nocardia farcinica* with104 sequences

On the 5th day following admission, considering the aggravation of the patient’s condition and sustained high fever, the lumber puncture was repeated. CSF examination showed a turbid CSF with 660 leukocytes/mm^3^, 2087 mg/L protein, 118.2 mmol/L chloride, 5.61 mmol/L glucose, and pressure was 400mmH2O. This time, the patient’s family agreed to perform CSF pathogen identification using mNGS. On the 7th day following admission, sputum smear indicated a Gram-positive bacilli and CSF mNGS analysis was suggestive of *Nocardia farcinica* with 104 sequence reads (Fig. 2). Together with medical history, imaging data and pathological findings, we concluded that the patient had pneumonia and brain abscess caused by *Nocardia farcinica*. Subsequently, we immediately amended the therapeutic regimen to Meropenem plus trimethoprim-sulfamethoxazole (TMP-SMX). Three days later, the level of consciousness, cough and sputum improved significantly. The temperature also obviously improved, fluctuating between 36.2 and 37.8 °C. Considering that it is difficult to afford high medical costs, the family chose to transfer the patient to the local hospital for treatment. Thereafter, we lost contact with the patient’s family.

## Sample collection and data analysis

Approximately 3–5 mL of CSF was collected and sealed using a sterile technique, shipped on dry ice to IngeniGen XunMinKang Biotechnology Inc. in China to perform mNGS detection. This test uses the high-throughput sequencing technology of illumina, USA. We perform metagenomic analysis on the microbial DNA sequences in the samples, compare them with the microbial nucleic acid sequences in the database, and then identify the pathogenic microorganisms. We used a double-ended 75 bp illumine nextseq platform for sequencing and detected a total of 9,597,156 * 2 sequences (which can be understood as a data volume of approximately 19 M) for this sample. The database used was provided by kraken. The range of detection covers the currently known pathogens including 7044 bacterial, 9233 viral, 2890 fungal, 172 parasites, 139 mycoplasma, 128 chlamydia, 102 rickettsia and 635 mycobacteria. The lowest detection limit was 100 copies/ml, and the specificity was greater than 99.6%. Of these, 104 reads uniquely aligned to the *Nocardia farcinica* genome.

## Discussion and conclusion

*Nocardia* is an opportunistic pathogen that can be seen everywhere in nature [1]. *Nocardia* can cause local or systemic infections by seeding in the lung through the respiratory tract or can enter the bloodstream through skin wounds. The disease can occur in healthy people but is more common in patients with diminished or defective immune function [1, 2]. In the study, we report a case with brain abscess caused by *Nocardia farcinica* diagnosed using mNGS of the CSF. The case with idiopathic thrombocytopenic purpura received oral methylprednisolone 5 mg/QD for 6 years and had risk factors for the disease. It is reported that the CNS infection of *Nocardia farcinica* is extremely rare [5]. Because *Nocardia* disease has no specific imaging and histopathological features [6], its diagnosis mainly relies on bacterial culture [7]. However, due to the long time and low positive rate of bacterial culture, it is easy to misdiagnose the infection. Additionally, lung CT and head MRI of the patient showed space-occupying lesions, which were more likely to be misdiagnosed as pulmonary tumor metastasis to the brain. Multiple sputum and CSF cultures revealed no *Nocardia*. As a result, the patient was misdiagnosed several times after onset.

The clinical manifestations of *Nocardia* infection in the lung are similar to those of other infectious diseases, with acute or subacute onset. Imaging findings are manifested as lobular or lobar pneumonia, which is prone to a chronic course in the future [8]. *Nocardia* can spread to any organ, particularly the CNS and even tends to recur [9]. Although *Nocardia* infection has no typical signs and symptoms, any patients with brain, soft tissue, or skin lesions and concurrent or recent lung diseases should be suspected of the disease. The present case had pulmonary infection followed by secondary CNS infection, so infection with the same pathogen needs to be considered. Because the disease is rare in clinical practice, insufficient understanding of this disease by clinicians can easily cause it to go undetected or misdiagnosed, resulting in undesirable patient outcomes.

*Nocardia* brain abscess is the characteristic focus of CNS infection, accounting for 1% ~ 2% of all intracranial abscesses [10, 11]. Among all acterial brain abscesses, *Nocardia farcinica* brain abscess has a high mortality rate, as high as 55% in immunocompromised patients. The high mortality is mainly attributed to the severity of underlying disease, difficulties in identifying the pathogen, and its inherent resistance to antibiotics, leading to inappropriate or late initiation of therapy [12].

Early diagnosis and initiation of therapy is key to reduction of Nocardia-associated mortalities and achieving desirable outcomes. According to reports, antibiotics including sulfa drugs, aminoglycosides, carbapenems, quinolones and tetracyclines are effective against *Nocardia* [13]. In general, long-term trimethoprim-sulfamethoxazole is the first choice. However, there are more and more reports of resistance to sulfa drugs [1]. Therefore, the combined use of two or more different kinds of antibiotics may be an ideal choice. In this case, we used anti-infective regimens such as methylene and vancomycin. Due to the short hospital stay, the treatment effect was not significant in the patient at an early stage.

There are many detection methods for *Nocardia*, but the positive rate of conventional detection methods for confirming *Nocardia* infection is low [14]. Novel mNGS testing of CSF can help clinicians diagnose and select antibiotics relatively quickly compared to conventional methods [10]. The powerful functionality and practical feasibility of mNGS enable its use for rapid detection of unknown pathogens, especially in immunocompromised patients with chronic infections.

## Data Availability

The data that support the findings of the current study are available from the corresponding author upon reasonable request.

## Abbreviations

CSF: Cerebrospinal fluid
mNGS: Metagenomics next-generation sequencing
MRI: Magnetic resonance imaging
TMP-SMX: Trimethoprim sulfamethoxazole
CNS: Central nervous system

## References

[1] Conville PS, Brown-Elliott BA (2017). The complexities of Nocardia taxonomy and identification. J Clin Microbiol.

[2] Steinbrink J, Leavens J, Kauffman CA, Miceli MH (2018). Manifestations and outcomes of nocardia infections: comparison of immunocompromised and nonimmunocompromised adult patients. Medicine (Baltimore).

[3] Kumar VA, Augustine D (2014). Nocardia farcinica brain abscess: epidemiology, pathophysiology, and literature review. Surg Infect.

[4] Peters BR, Saubolle MA, Costantino JM (1996). Disseminated and cerebral infection due to Nocardia farcinica: diagnosis by blood culture and cure with antibiotics alone. Clin Infect Dis.

[5] Valdezate S, Garrido N, Carrasco G, Medina-Pascual MJ, Villalón P, Navarro AM, Saéz-Nieto JA (2017). Epidemiology and susceptibility to antimicrobial agents of the main Nocardia species in Spain. J Antimicrob Chemother.

[6] Rouzaud C, Rodriguez-Nava V, Catherinot E, Méchaï F, Bergeron E, Farfour E, Scemla A, Poirée S, Delavaud C, Mathieu D, Durupt S, Larosa F, Lengelé JP, Christophe JL, Suarez F, Lortholary O, Lebeaux D (2018). Clinical assessment of a Nocardia PCR-based assay for diagnosis of Nocardiosis. J Clin Microbiol.

[7] Coussement J, Lebeaux D, van Delden C, Guillot H, Freund R, Marbus S, Melica G, Van Wijngaerden E, Douvry B, Van Laecke S, Vuotto F, Tricot L, Fernández-Ruiz M, Dantal J, Hirzel C, Jais JP, Rodriguez-Nava V, Lortholary O, Jacobs F, European Study Group for Nocardia in Solid Organ Transplantation (2016). Nocardia infection in solid organ transplant recipients: a multicenter European case-control study. Clin Infect Dis.

[8] Zhu JW, Zhou H, Jia WQ, You J, Xu RX (2020). A clinical case report of brain abscess caused by Nocardia brasiliensis in a non-immunocompromised patient and a relevant literature review. BMC Infect Dis.

[9] Paige EK, Spelman D (2019). Nocardiosis: 7-year experience at an Australian tertiary hospital. Intern Med J.

[10] Huang T, Chen Y, Zhang J, He R, Qu D, Ye Q, Chen X (2021). Rapid and accurate diagnosis of brain abscess caused by Nocardia asiatica with a combination of Ziehl-Neelsen staining and metagenomics mNGS. Eur J Neurol.

[11] Hofmeister MG, Dao A, Young AM (2016). Worsening stroke symptoms in an 80-year-old man. JAMA Neurol.

[12] Mamelak AN, Obana WG, Flaherty JF, Rosenblum ML (1994). Nocardial brain abscess: treatment strategies and factors inflfluencing outcome. Neurosurgery.

[13] Lebeaux D, Bergeron E, Berthet J, Djadi-Prat J, Mouniée D, Boiron P, Lortholary O, Rodriguez-Nava V (2019). Antibiotic susceptibility testing and species identification of Nocardia isolates: a retrospective analysis of data from a French expert laboratory, 2010-2015. Clin Microbiol Infect.

[14] Anagnostou T, Arvanitis M, Kourkoumpetis TK, Desalermos A, Carneiro HA, Mylonakis E (2014). Nocardiosis of the central nervous system: experience from a general hospital and review of 84 cases from the literature. Medicine (Baltimore).

